# Efficacy of psychosocial interventions for survivors of intimate partner violence: protocol for a systematic review and meta-analysis

**DOI:** 10.1192/bjo.2022.625

**Published:** 2023-01-03

**Authors:** Hannah M. Micklitz, Carla M. Glass, Jürgen Bengel, Lasse B. Sander

**Affiliations:** Department of Medical Psychology and Medical Sociology, Faculty of Medicine, University of Freiburg, Freiburg, Germany; Department of Rehabilitation Psychology and Psychotherapy, Institute of Psychology, University of Freiburg, Freiburg, Germany

**Keywords:** Intimate partner violence, domestic violence, psychosocial interventions, meta-analysis, review

## Abstract

Survivors of intimate partner violence (IPV) are at risk for serious health consequences, and providing effective psychosocial interventions to support these individuals is a major global health challenge. Previous systematic reviews and meta-analyses in this field do not allow for clear conclusions about the efficacy of these interventions, owing to a narrow focus on specific subpopulations or intervention formats. This protocol presents a systematic review and meta-analysis, which will provide a comprehensive overview of the empirical evidence of various psychosocial interventions for survivors of IPV and investigate their efficacy in improving safety-related, mental health and psychosocial outcomes both overall and within homogeneous subgroups (trial registration: https://osf.io/4gp95). We will systematically search the literature databases PsycInfo, MEDLINE, Embase and CENTRAL. Randomised controlled trials evaluating the efficacy of psychosocial interventions in increasing the safety or mental health of IPV survivors compared with a control group will be eligible. We will extract relevant data from eligible studies and assess study quality using the Cochrane Risk of Bias 2 (RoB 2) tool. We will qualitatively summarise the results and we will calculate weighted effect sizes under random effect model assumption for the primary outcomes IPV, depression and post-traumatic stress disorder. We will perform subgroup analyses to investigate the moderating effects of theoretical basis, delivery mode, intensity and setting of psychosocial interventions. The resultant overview of the current body of evidence for psychosocial interventions for IPV survivors is intended to inform future research and practice.

Intimate partner violence (IPV) is a highly prevalent and global concern of public health and a major human rights violation. Globally, it is estimated that around 26% (95% CI 22–30%) of women have been subjected to physical and/or sexual violence from a current or former male intimate partner at some stage in their lives.^1^ IPV is considered the most widespread manifestation of violence against women,^1^ yet IPV affects people of all genders, regardless of sexual orientation or relationship type.^2^ Survivors of IPV are at an elevated risk for loss of home,^3^ economic insecurity and unemployment^4^ and deterioration in quality of life^5^ and social functioning.^6^ Further, experiencing IPV increases the risk for numerous mental health conditions, including post-traumatic stress disorder (PTSD), depression, anxiety, substance misuse, pain syndromes, sleep disturbances and suicidality.^6,7^ In its most severe form, IPV can escalate into intimate partner homicide.^8^

Effective and low threshold support services are urgently needed to improve the safety of IPV survivors and to reduce harms and negative consequences they face. For this purpose, a broad range of psychosocial interventions have been developed and implemented in healthcare and support systems worldwide.^9^ These interventions include counselling, professional and peer support services, psychotherapy and more comprehensive programmes.^10^ Advocacy and cognitive–behavioural approaches are commonly used,^11,12^ sometimes integrated into a hybrid treatment model.^13^ The psychosocial interventions can be short or long term^13^ and take place in primary care, crisis centres, shelters, in- and out-patient settings and in the community,^14^ they can be delivered face-to-face individually, for couples or in groups,^15–17^ remote via telephone or video calls^18,19^ and online through internet and smartphone-based platforms and applications that contain human guidance in varying degrees.^18,20,21^

Previous systematic reviews and meta-analyses found only scarce and inconsistent evidence on the efficacy of psychosocial interventions in preventing recurrence of IPV and reducing related health and psychosocial impairment of IPV survivors. Many authors have pointed out the poor quality of primary studies, mostly because of pre–post study design,^22^ small samples^23^ and unreliable outcomes measures.^21,22^ However, the reviews and meta-analyses are also affected in their scope and reliability by methodological limitations. Most reviews and meta-analyses exclusively focus on specific subpopulations (e.g. pregnant women,^24,25^ LGTBQ+ couples^26^), settings (e.g. during or after shelter,^27^ healthcare settings^9,14,28^), intervention formats (e.g. digital interventions,^18,20,21,29^ advocacy interventions,^22,30^ psychological interventions^16,31^) or outcomes (e.g. reduction of IPV,^17^ social support and mental health outcomes^19^). Although these approaches recognise the wide heterogeneity of existing psychosocial interventions, their use results in difficulties in interpreting the overall effectiveness and the comparative effectiveness across homogeneous subgroups. Moreover, it also impedes investigations into potential moderators of the effectiveness of psychosocial interventions for IPV survivors. Trabold and colleagues have attempted to address this challenge in a broad systematic review.^23^ However, it included only studies up to 2016 and did not perform meta-analytic pooling.

Therefore, in this protocol we present a systematic review and meta-analysis that will integrate a broad range of psychosocial interventions for survivors of IPV to achieve the following research objectives:

- provide an up-to-date and comprehensive overview of psychosocial interventions for IPV survivors
- perform meta-analyses investigating the efficacy of psychosocial interventions for survivors of IPV in terms of safety-related, mental health and psychosocial outcomes
- perform subgroup analysis to reduce heterogeneity and to investigate potential effect moderators.

## Method

The reporting of this protocol follows the Preferred Reporting Items for Systematic Review and Meta-Analysis Protocols (PRISMA-P) statement. We will report the systematic review and meta-analysis in accordance with Preferred Reporting Items for Systematic Reviews and Meta-Analyses (PRISMA) guidelines. We preregistered the review in the Open Science Framework (OSF) database (https://osf.io/4gp95). We will list potential deviations from the study protocol in the final report.

### Eligibility criteria

Studies will be selected based on the population, intervention, comparison, outcomes and study design (PICOS) criteria listed in the Appendix below. We will include studies published in a peer-reviewed journal, irrespective of publication date or language. If necessary, publications will be translated to English.

The target population consists of IPV survivors (i.e. people exposed to IPV prior to or at study baseline). Samples can include individuals or couples, but samples including only perpetrators are ineligible. Studies will qualify for inclusion if they investigate any psychosocial intervention that explicitly targets IPV or the mental health of IPV survivors, regardless of setting, delivery mode and duration. We will exclude studies that examine interventions for primary prevention (e.g. interventions targeting stigma, screening-only interventions), interventions aimed only at perpetrators or gatekeepers (e.g. family doctors, teachers), interventions that target exclusively associated symptoms (e.g. safe sex) or economic well-being (e.g. cash transfer), or provide physical safety or medical treatment only (e.g. shelter, emergency department). Studies with any comparison group (e.g. no treatment, waiting list, placebo, treatment as usual (TAU) or another treatment) are eligible. Studies need to report a quantitative measure related to IPV, mental health or psychosocial well-being. In terms of study design, only randomised controlled trials will be included. We will record study protocols to provide an overview on upcoming research.

### Search strategy and selection process

We will systematically search the electronic databases PsycINFO (via EBSCO), Medline (via PubMed), Embase (via Ovid) and the Cochrane Central Register of Controlled Trials (CENTRAL). We will use a predefined search string, consisting of different medical subject headings (MeSH terms) and text words related to the key concepts of IPV, intervention/treatment and randomised controlled trial (RCT) (Supplementary Appendix A, available at https://dx.doi.org/10.1192/bjo.2022.625). We tested the sensitivity of the search string in a pilot search with a validation set of 25 relevant studies and obtained a coverage of 100%. References yielded by the systematic database search will be uploaded to the Cochrane toolkit Covidence.^32^ This software assists the selection and data collection process and thus improves accuracy of the procedure. After removal of duplicates, we will screen titles and abstracts. If the contained information indicates that studies are ineligible, they will be declined. Otherwise, full texts will be screened. Two reviewers (H.M.M., C.M.G.) will conduct this procedure independently. Divergent decisions about the inclusion of a study will be resolved via consensus discussion or by consultation with a third reviewer (L.B.S.). We will hand-search reference lists of selected articles (backward search) to identify eligible trials not detected by the electronic database searches. In addition, forward searches will be performed using the Google Scholar platform. We will use a PRISMA flowchart to illustrate the selection process and to specify reasons for study exclusion.

### Ethics statement

No ethics approval will be needed as only data from previously published studies will be analysed.

### Data items

We will extract the data using a predefined and piloted extraction sheet (Supplementary Appendix B). For each included study, the following items will be extracted from publications: (a) study identification items, (b) study design characteristics, (c) participant characteristics, (d) intervention characteristics, (e) control characteristics and (f) characteristics and results for safety-related, mental health, psychosocial, satisfaction, acceptance and treatment adherence outcomes. Data will be extracted by one researcher (C.M.G.) and double-checked by a second researcher (H.M.M.). A third researcher (L.B.S.) will be consulted in case of ambiguities.

### Quality assessment

We will evaluate risk of bias using the Cochrane Risk of Bias 2 (RoB 2) tool for RCTs.^33^ For each primary study we will assess bias in five domains: (1) bias arising from the randomisation process, (2) bias due to deviations from the intended interventions, (3) bias due to missing outcome data, (4) bias in measurement of the outcome, and (5) bias in selection of reported results. For domain 2, we will judge the ‘effect of the assignment to the intervention’. Based on judgement across these five domains, we will rate overall risk of bias. We will determine likelihood of publication bias by identification of study protocols and trial registrations. If the number of included studies is sufficient, publication bias will also be evaluated graphically by funnel plot and statistically by Egger's test of funnel plot asymmetry.^34^ Additionally, we will rate the quality of evidence for each primary outcome using the Grading of Recommendations Assessment, Development and Evaluation (GRADE) guidelines.^35^ The evaluation criteria are (a) risk of bias, (b) inconsistency, (c) indirectness, (d) imprecision and (e) publication bias. Two reviewers (C.M.G., H.M.M.) will independently perform the quality assessment. Any deviations will be resolved by discussion; if needed, a third reviewer (L.B.S.) will be included.

### Data synthesis

#### Qualitative synthesis

We will conduct a narrative synthesis of all included studies. We will report characteristics and results (see ‘Data items’ above) of each included study in both text and tables. We will summarise study protocols and report ongoing research in the field.

#### Meta-analysis

We will calculate between-group effect sizes (Hedges’ *g* and standard error) for each included study using post-intervention scores (i.e. first measurement of outcomes after treatment exposure) for the intervention and control groups. Additionally, we will use available follow-up scores to examine whether the effects persist at medium-term follow-up (i.e. 6–11 months post baseline) and long-term follow-up (i.e. ≥12 months post baseline).

We will perform meta-analyses calculating pooled between-group effect sizes (Hedges’ *g* with 95% confidence intervals for continuous outcomes or odds ratio for dichotomous outcomes) for psychosocial interventions compared with control conditions for each outcome in a random effects model. Weighting of studies will be based on inverse variance. Besides visual inspection of the forest plot, the *I*^2^ statistic will be used to examine statistical heterogeneity of studies. Heterogeneity will be considered to be low at 0–40%, moderate at 30–60%, substantial at 50–90% and considerable at 75–100%.^33^ We will interpret effect sizes following Cohen's suggestion (1992), in which a value of 0.2 indicates a small effect, 0.5 a medium effect and 0.8 a large effect. Statistical significance will be judged on *P* < 0.05.

Only studies with inactive control groups (i.e. no treatment, waiting list, TAU, placebo) that report outcomes on safety (frequency/intensity of IPV, danger assessment, safety-related behaviours), mental health (depression, PTSD, anxiety, psychological distress, suicidality or substance use) and/or psychosocial outcomes (perceived self-efficacy, personal resources, social support, decisional conflict, empowerment, quality of life) will be included in the meta-analysis. Further, only studies providing sufficient values (e.g. means, s.d., s.e.) to compute effect sizes for total scores will be eligible for analysis, since estimates rely on published data only. If a study reports multiple scales for the same outcome, the values for the measure most frequently applied will be included to keep heterogeneity as low as possible. If available, intention-to-treat data (ITT) will be used. We will perform a meta-analysis if at least three of the included studies report data on the same outcome.

We will use Review Manager version 5.4 (RevMan 5.4)^36^ to conduct the analyses.

#### Subgroup analysis

We will investigate predefined intervention and study characteristics as potential effect moderators. We will compare types of theoretical basis (presumably, advocacy versus psychological versus integrative interventions), types of intervention delivery mode (face-to-face versus digital interventions), types of intervention format (individual versus group), different treatment intensities (low-intensity interventions with 1–3 sessions versus medium-intensity interventions with 4–9 sessions or ≥6 months access to websites/apps versus high-intensity interventions with ≥10 sessions), treatment settings (shelter versus community versus clinical setting), resources of treatment settings (low- and middle-income countries versus low-resource settings in high-income countries versus high-income countries) and characteristics of inactive control conditions (TAU versus waiting list). Subgroup analysis will be feasible if three or more studies per subgroup are available.

#### Sensitivity analysis

We will conduct sensitivity analysis by excluding trials at high risk of bias and by excluding outliers.^33^

## Discussion

Effective and safe psychosocial interventions for survivors of IPV are required to prevent recurrence of violence and reduce negative health and psychosocial sequelae. With this systematic review and meta-analysis, we will provide a comprehensive overview of the current evidence base on various psychosocial interventions for IPV survivors. By integration of diverse populations, settings, interventions and outcomes, as well as inclusion of recently published studies, we will address limitations of previous systematic reviews and meta-analyses. We will quantitatively analyse the efficacy of psychosocial interventions for IPV survivors in improving safety and reducing related health and psychosocial impairment. Further, by performing subgroup analyses, we will investigate intervention and study characteristics that moderate the effectiveness of psychosocial interventions for survivors of IPV. Doing so, we aim to identify effective intervention approaches and promising lines of research. However, aside from these strengths, there are potential limitations that might affect the findings of this review. First, studies in the outlined research area are known to be of poor quality,^22,23,37^ which is likely to distort our effect estimates.^38^ To detect bias potentially arising from methodological quality, we will perform sensitivity analyses, where only high-quality studies will be included. Second, because of the broad scope of this review, we expect substantial heterogeneity across studies, which reduces the confidence in our quantitative findings. We will address heterogeneity with subgroup analysis and reasonable selection of studies included in the meta-analyses.

## Data Availability

Data availability is not applicable to this article as no new data were created or analysed in its preparation.

## Supplementary material

For supplementary material accompanying this paper visit http://doi.org/10.1192/bjo.2022.625. click here to view supplementary material

## Appendix

### Participants

Individuals or couples with prior or current experience of IPV

Exclusion criteria: perpetrators (only)

### Intervention

- Interventions that explicitly target IPV, mental health and/or psychosocial outcomes
- No restrictions on delivery mode (individual, couple, group, telephone, digital with or without guidance)
- No restrictions on setting (e.g. healthcare, community, shelter or refuge)
- No restriction on duration/intensity

Exclusion criteria:

- Interventions for primary prevention of IPV (e.g. interventions targeting stigma, screening only)
- Interventions designed for perpetrators only
- Interventions designed for gatekeepers (e.g. family doctors, teachers)
- Interventions targeting economic well-being only (e.g. cash transfer)
- Interventions targeting associated symptoms only (e.g. safe sex)
- Interventions providing physical safety or medical treatment only (e.g. shelter, refuge, crisis centre)

### Comparator

- Treatment as usual (TAU) (e.g. standard service care, informational session, unstructured counselling, routine prenatal, shelter or HIV care, informational websites)
- Another treatment
- Placebo (e.g. attention control, educational interventions on related topics)
- Waiting list
- No treatment

### Outcomes

Any quantitative measure related to:

- IPV (e.g. occurrence and frequency of IPV, safety-related behaviours)
- Mental health (e.g. symptoms of depression, anxiety, PTSD, substance misuse, suicidal ideation, general mental health)
- Psychosocial outcomes (e.g. quality of life, social functioning, self-esteem, self-efficacy, decisional conflict, social support, general distress)

### Study design

- Randomised controlled trials
- Study protocols
